# Improved Diagnosis in Children with Partial Epilepsy Using a Multivariable Prediction Model Based on EEG Network Characteristics

**DOI:** 10.1371/journal.pone.0059764

**Published:** 2013-04-02

**Authors:** Eric van Diessen, Willem M. Otte, Kees P. J. Braun, Cornelis J. Stam, Floor E. Jansen

**Affiliations:** 1 Rudolf Magnus Institute of Neuroscience, Department of Pediatric Neurology, University Medical Center Utrecht, Utrecht, The Netherlands; 2 Biomedical MR Imaging and Spectroscopy Group, Image Sciences Institute, University Medical Center Utrecht, Utrecht, The Netherlands; 3 Department of Clinical Neurophysiology, VU University Medical Center, Amsterdam, The Netherlands; University Medical Center Groningen UMCG, The Netherlands

## Abstract

**Background:**

Electroencephalogram (EEG) acquisition is routinely performed to support an epileptic origin of paroxysmal events in patients referred with a possible diagnosis of epilepsy. However, in children with partial epilepsies the interictal EEGs are often normal. We aimed to develop a multivariable diagnostic prediction model based on electroencephalogram functional network characteristics.

**Methodology/Principal Findings:**

Routinely performed interictal EEG recordings at first presentation of 35 children diagnosed with partial epilepsies, and of 35 children in whom the diagnosis epilepsy was excluded (control group), were used to develop the prediction model. Children with partial epilepsy were individually matched on age and gender with children from the control group. Periods of resting-state EEG, free of abnormal slowing or epileptiform activity, were selected to construct functional networks of correlated activity. We calculated multiple network characteristics previously used in functional network epilepsy studies and used these measures to build a robust, decision tree based, prediction model. Based on epileptiform EEG activity only, EEG results supported the diagnosis of with a sensitivity and specificity of 0.77 and 0.91 respectively. In contrast, the prediction model had a sensitivity of 0.96 [95% confidence interval: 0.78–1.00] and specificity of 0.95 [95% confidence interval: 0.76–1.00] in correctly differentiating patients from controls. The overall discriminative power, quantified as the area under the receiver operating characteristic curve, was 0.89, defined as an excellent model performance. The need of a multivariable network analysis to improve diagnostic accuracy was emphasized by the lack of discriminatory power using single network characteristics or EEG's power spectral density.

**Conclusions/Significance:**

Diagnostic accuracy in children with partial epilepsy is substantially improved with a model combining functional network characteristics derived from multi-channel electroencephalogram recordings. Early and accurate diagnosis is important to start necessary treatment as soon as possible and inform patients and parents on possible risks and psychosocial aspects in relation to the diagnosis.

## Introduction

Epilepsy is a common neurological disorder, yet, accurate diagnosis and classification at an early stage still poses a challenge to the clinician. The diagnosis of epilepsy is primarily based on the clinical history and may be supported by information provided by interictal EEG recording. For proper classification of the epilepsy syndrome the EEG is indispensable. Additional neuroimaging or sleep deprivation EEG is often used when the initial clinical diagnosis is not conclusive or when more information is needed for classification of the epilepsy syndrome and assessment of prognosis. Clinical diagnosis has a high interobserver variation. One study found that 25 percent of patients were incorrectly diagnosed as having had a seizure at the initial presentation [1]. In addition, evaluation of EEG abnormalities may be subjective [2] and is not very sensitive. For example, epileptiform activity is demonstrated only in 29 to 55 percent of patients on routinely performed EEG recordings [3]. To a lesser extent, EEG abnormalities may also be found in healthy controls, especially in children [4]. Together, interictal EEG recordings are supportive but often not conclusive in the initial clinical diagnosis of epilepsy. Developing EEG measurements with increased sensitivity and specificity would be highly valuable in the early clinical diagnosis of epilepsy. Accurate diagnosis at an early stage may allow more rapid optimization of treatment and improve counselling with regard to lowering risks with necessary life rules and restrictions. Particularly children with partial epilepsy could benefit from early and accurate diagnosis, since interictal epileptiform EEG activity is often absent [4], possibly causing a delay in diagnosis and decision-making.

In this study we used the expanding knowledge on functional neural network organization in normal and diseased brain [5]. Brain network organization is typically summarized using multiple network characteristics such as global efficiency, local clustering, power-law degree distribution and centrality measures [6]. Recently, it has been shown that interictal functional network characteristics differ between controls and patients with chronic partial epilepsy [7], [8], [9], [10], [11].

We aimed to explore the clinical value of functional network characteristics by investigating network characteristics separately and combined in a multivariable diagnostic prediction model. We hypothesized that functional network characteristics enhance sensitivity and specificity of diagnosis of partial epilepsy in children at initial assessment.

## Materials and Methods

### Patients

Children referred, between January 2006 and December 2010, to the outpatient department of pediatric neurology, University Medical Center Utrecht, The Netherlands after one or more suspected epileptic event(s) were eligible for our study. We included children who were eventually diagnosed with new onset partial epilepsy. Children with neurological or psychiatric co-morbidities, including developmental delay, were excluded. The clinical diagnosis of epilepsy was defined by at least two unprovoked seizures within one year, judged by two neurologists to be of epileptic origin. The clinical diagnosis was supported in a subset of patients by epileptiform abnormalities (interictal epileptiform discharges (IEDs) such as sharp waves, (poly) spikes or (poly) spike-wave complexes or abnormal slowing), on routinely performed EEG. In patients clinically diagnosed with epilepsy but with a normal routine EEG recording, the diagnosis was confirmed by subsequent sleep deprivation EEG recordings, neuroimaging or clinical follow-up with history of more highly suspected events. An MRI was performed in all children diagnosed with epilepsy, not classified as idiopathic focal epilepsy. Epilepsy was excluded in the control group, based on clinical history, EEG results, and at least one year of uneventful follow up. This control group was individually matched with the patient group on gender and age. Neither patients nor controls had a history of febrile seizures, generalized epilepsy, or were on (chronic) anticonvulsive medication.

The institutional ethical committee approved the study and concluded that the Dutch Medical Research Involving Human Subjects Act did not apply, and written informed consent was not required.

### Data acquisition and selection

Routinely performed interictal EEG recording was available for each child. Interictal EEGs were recorded according the international 10–20 system (SystemPlus Evolution, Micromed) in awake and eyes closed (resting-state) condition against an average reference electrode. Impedance of each electrode was kept below 5 kΩ. Data was high- and low-pass filtered at 0.16 and 70 Hz, respectively. Sampling frequency was 512 Hz.

All EEG recordings contained 21 scalp electrodes (F8, F4, Fz, F3, F7, T8, C4, Cz, C3, T7, P8, P4, Pz, P3, P7, O1, O2, Fp1, Fp2, A1, and A2) and were visually inspected (EvD). To assure stable EEG brain dynamics for the calculation of network characteristics, for each subject we selected four epochs (eight seconds each) at the beginning of the interictal EEG recording [12], [13]. All epochs were free of IEDs, abnormal slowing, and electrocardiographic or motion-induced artifacts. Two frontoparietal and basal temporal electrodes (Fp1, Fp2, A1, and A2) were excluded to minimize eye-movement artifacts. The epochs were independently re-inspected by a clinical epileptologist (FEJ) on artifacts and IEDs. Finally, all selected EEG epochs were converted to ASCII files to enable functional network analysis. All data were additionally filtered to obtain standard broadband frequencies in the range of 0.5 to 45 Hz.

To check whether subjects with partial epilepsy could be distinguished from controls by means of spectral analysis, we computed the relative and absolute power spectral densities averages over epochs and subjects [14].

### Computation of functional network characteristics

Individual functional EEG networks were constructed for each subject using their broadband filtered data. Functional network organization is based on the relatively new concept of functional connectivity. The statistical interdependencies for each pair of EEG electrode time series are considered as functional connectivity and used to construct a functional network per subject for each of the four epochs and were averaged per subject. Multiple complementary methods exist to estimate the statistical interdependency between two time series [15]. We based our functional network construction on a functional connectivity index that was previously applied in epilepsy studies, namely the synchronization likelihood (SL) [13], [16], [17], [18]. SL detects both linear and nonlinear dependencies between the time series and is considered to be a measure of generalized synchronization [19]. The SL expresses the functional connectivity as a value between 0 and 1, and allows the construction of weighted functional networks (i.e., the connectivity strength between two electrodes is preserved) [20]. Weighted SL networks were constructed using the freely available BrainWave software (http://home.kpn.nl/stam7883/brainwave.html).

We have chosen to include network characteristics used to study functional networks in epilepsy and published recently in neuroscience papers (for review see [6]). For each weighted network, we calculated the following characteristics: weighted degree centrality (strength), weighted shortest path length, weighted clustering coefficient, weighted betweenness centrality, weighted closeness centrality and weighted eigenvector centrality. An additional characteristic was the powerlaw distribution index [21], [22]. Apart from mean values of each network characteristic across all network nodes, we calculated both minimal and maximal values to increase the information used for building a multivariable diagnostic prediction model. The mathematical forms of the network characteristics are provided in the next paragraph.

### Mathematical forms of the network characteristics

#### Network construction

For each of the 70 subjects we constructed a weighted undirected network, described by the graph *G* = (*N*, *W*), where *N* is the set all 17 EEG electrodes and 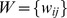
is the *N*×*N* symmetric weight matrix, where *w_ii_* = 0 and *w_ij_* the synchronization likelihood index determined between electrode *i* and *j* [19].

#### Weighted degree centrality (strength)

Four network characteristics of edge centrality were included in the model, which determined the relative importance of a node within the graph [23]. The weighted degree centrality, or strength for node *i* was defined as
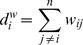



The minimal, mean and maximal strength were defined as
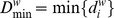


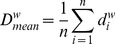


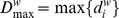



#### Weighted shortest path length

For a given node *i* in the graph, the shortest path algorithm finds the path with lowest cost (i.e. the shortest path length) between that node and every other node. For the weighted shortest path length, the path between two nodes *i* and *j* is find by minimizing the sum of weights assigned to the edges on their path. The average shortest path length for node *i* to all other nodes is defined as
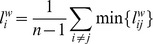



Here, 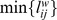 is the weighted shortest path length between node *i* and *j*. We considered high values of the synchronization index as close functional distance and low values of the synchronization index as large functional distance (i.e. 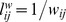). In our dataset no disconnected nodes were present. The minimal, mean and maximal weighted shortest path were defined as
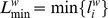


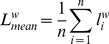


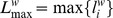



#### Weighted closeness centrality

Edges that have short distances to other edges have high closeness; this principle is used in the calculation of the weighted closeness centrality. Formally
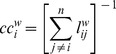



The minimal, mean and maximal weighted closeness centrality were defined as
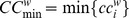


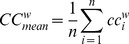


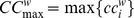



#### Weighted betweenness centrality

The weighted betweenness centrality relies on the identification of the number of weighted shortest paths that pass through a node. The more passages the higher the betweenness centrality. The weighted betweenness centrality is defined as
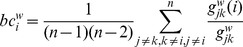



where 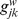 is the shortest path between two nodes and 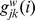 is the number of those nodes that pass through node *i*. The mean and maximal weighted betweenness centrality were defined as
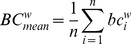


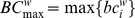



#### Eigenvector centrality

Eigenvector centrality is based on the greatest eigenvector of the weight matrix *W* [24]. The eigenvectors of the weight matrix indicate the nodes that show high connections with most other nodes. In contrast to the weighted degree centrality, it specifically favors nodes that are connected to nodes that are themselves central within the network [25]. If 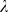 is the largest eigenvalue and *ec^w^* the corresponding eigenvector, then 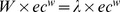 or similar 
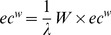
 and 
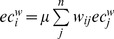
, where 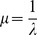 is the proportionality factor so that 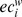 is proportional to the sum of connectivity scores of all nodes connected to it. We used the minimal, mean and maximal values of the eigenvector in our model, defined as
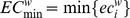


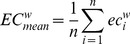


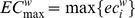



#### Weighted clustering coefficient

The clustering coefficient is a measure of degree to which nodes in a graph tend to cluster together. We used the weighted clustering coefficient [26], [27]. The weighted clustering coefficient for each node *i* was defined as
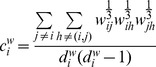



and takes into account the weights of all edges in a triangle, excluding weights not participating in any triangle. The minimal, mean and maximal weighted clustering coefficient were defined as
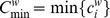


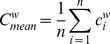


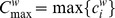



#### Power law scaling index

The collection of (weighted) degree centrality measures 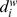 in a functional brain network often forms a distribution, *p(d)*, that decays as a power law [28]. This power law decay ranges from a minimal value 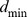 to the maximal value, and scales with the index *α* as 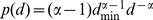. The power law scaling index *α* was one of the network characteristics included in the prediction model. The calculation of 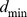
and *α* was done by a robust maximum likelihood method [21].

### Model development

The diagnostic prediction model was built with available software packages in the open-source R environment [29], [30]. The predictive diagnostic model has been based on a robust ensemble algorithm, namely the random forest classifier [31] using a free implementation provided as the random forest package [32] The core of the random forest classifier is the binary decision tree, a data type that stores elements hierarchically in nodes. Each decision tree is grown on different bootstrapped sample collections (i.e., randomly drawn instances with replacement from the original dataset) on a randomly selected subset of all available predictors. The random selection of predictors increases the generalizability of the individual decision trees, whereas the collection of multiple decision trees in one forest increases model performance [31].

For our data, a subset of 5 random predictors for each decision tree was found to give highest accuracy. After building the random forest diagnostic prediction model, we assessed the ability to differentiate between subjects with and without partial epilepsy using the area under the Receiver Operating Characteristic (ROC) curve (AUC) [33]. An AUC higher than 0.8 reflects excellent discrimination [34]. However, the AUC is typically considered to be too optimistic when the diagnostic model is tested on the same data that is used to build the model. Internal validation methods correct for this. We used the regular bootstrapping approach for internal validation, which is the preferred method when dealing with relative small datasets [35], [36]. With this method, the model is rebuild on multiple random samples drawn with replacement from the full dataset. In this study we only report the bootstrap corrected results.

### Statistical analysis

Group differences on all network characteristics were individually assessed with the independent Student T-test. Each diagnostic prediction model was fit on 1000-bootstrapped realization of the original dataset. We calculated a bootstrap corrected average ROC curve and corresponding average AUC, sensitivity, specificity, positive predictive and negative predictive values. All statistical analyses were performed in R using the pROC [37] package [29].

## Results

### Patient characteristics

A total of 419 children visited the outpatient department of pediatric neurology, between January 2006 and December 2010, after a recent event of possible epileptic origin. In total, 75 children were diagnosed with generalized epilepsies, 69 with partial epilepsy and 38 with febrile seizures. In 52 cases, diagnosis remained undetermined but clinical follow-up revealed no event(s). Of the 69 patients who were eventually diagnosed with partial epilepsy, only 35 patients both met our strict inclusion criteria and had an EEG recording of sufficient quality for functional network analysis (11 girls and 24 boys, mean age 10.1±3.4 years). In 185 children, epilepsy was excluded and an alternative clinical diagnosis was made for the paroxysmal event(s). From this group, 35 controls were selected; individually match on age and gender with the patient group (mean age 9.9±3.1 years). Detailed clinical characteristics are provided as supplemental information (for patients *Table S1* and for controls *Table S2*). None of the children had paroxysmal event in the days prior or post EEG recording, thereby excluding pre- or postictal changes of the EEG signal [16]. Epileptiform interictal EEG activity was present in 77% (27 out of 35) of patients and supported clinical diagnosis. Subsequent sleep deprivation EEG was performed in 5 patients showing epileptiform activity in all cases. In the remaining 3 patients, the diagnosis of epilepsy was reconfirmed by clinical follow-up. The average number of seizures prior to presentation at the outpatient department was 5 (range: 1–36). The events of the children in the control group were eventually diagnosed as syncope (11 cases), behavioural/psychogenic events, including tic and stereotypy (11 cases), staring/non-attentiveness without epileptic origin (7 cases), pavor nocturnus (3 cases), arrhythmia (1 case), segmental non-epileptic myoclonic jerks (1 case) and breath holding spells (1 case). In the control group, aspecific EEG abnormalities were present in 9% (3 out of 35).

### Power spectrum and network characteristics

**Figure 1:**
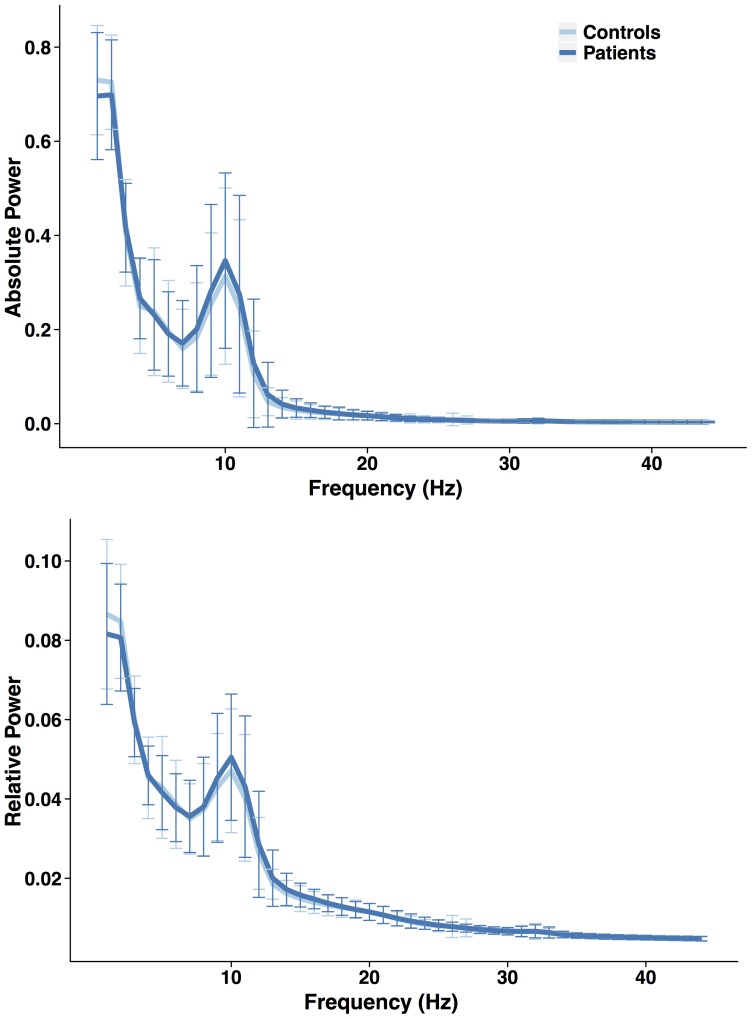
(power spectral density plots of patients and controls). Mean absolute and relative power spectral densities between 0 and 45 Hz averaged over epochs and subjects per group. Variation, defined as the standard deviation, is indicated by cross bars. Spectral densities between groups largely overlap.

**Figure 2:**
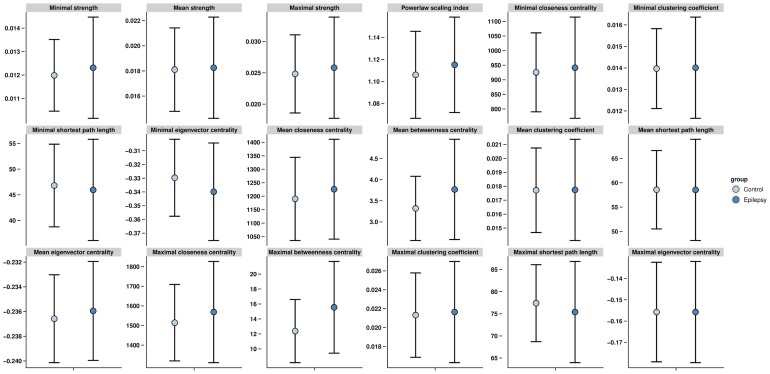
(individual network characteristics of patients and controls). Mean and standard deviation of all network characteristics, used in the predictive model, calculated for the broadband frequency. No significant differences were found for each characteristic between groups.

**Figure 3:**
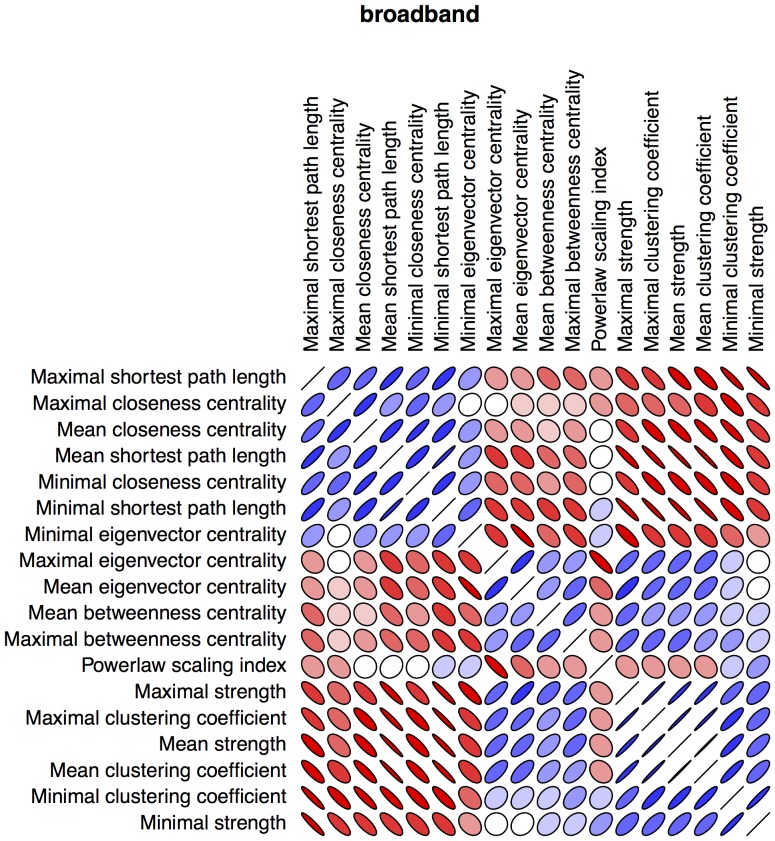
(correlation matrix included network characteristics). Pearson correlation matrix of all network characteristics for the broadband frequency. Red color indicates positive correlation, blue color indicates negative correlation. Tensor anisotropy indicates strength of correlation (0 correlation, circular; full correlation, single line).

Power spectral density revealed no differences between patients and controls (*Figure 1*). None of the individual network characteristics was significantly different between groups (*Figure 2*). The lack of discriminatory power in the frequency analysis and individual network characteristics emphasized the need for a multivariable model. The network characteristics used in the multivariable model contained unique information, as shown in our correlation matrix (*Figure 3*).

### Diagnostic model

**Figure 4:**
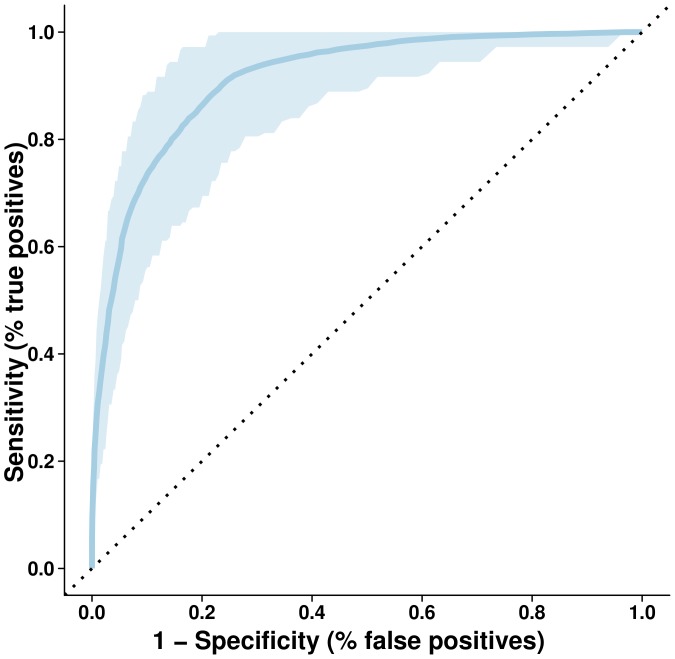
(ROC curve). ROC curve (dark blue) and 95% confidence interval for the network characteristics based on the broadband frequency.

The ROC curve for the predictive model using broadband network characteristics is shown in *Figure 4*. The model had a mean sensitivity of 0.96 [95% confidence interval (CI): 0.78 – 1.00], mean specificity of 0.95 [CI: 0.76 – 1.00], mean positive predictive value of 0.96 [CI: 0.82 – 1.00] and a mean negative predictive value of 0.96 [CI: 0.81 – 1.00]. The model performance was excellent, with an AUC of 0.89 [CI: 0.80 – 0.95]. We found similar results in terms of model performance if the analysis was repeated for specific frequency bands including delta band (0.5–4 Hz), theta band (4–8 Hz), alpha band (8–12 Hz), beta band (12–30 Hz) and gamma band (30–45 Hz) (data not shown).

In an additional sub analysis, we bootstrap validated the model specifically using only the subset of 8 epilepsy patients of whom routinely performed EEG recordings were judged normal, resulting in a sensitivity of 0.86 [CI: 0.64 – 1.00]. Similarly, we tested the subset of 3 controls in whom routinely performed EEG recordings contained aspecific abnormalities and in these patients the model was found to have a good performance as well with a specificity of 0.81 [CI: 0.33 – 1.00].

## Conclusions

In this study we were able to build a highly accurate diagnostic prediction model to distinguish children with partial epilepsy from children who were judged to have had non-epileptic events, with network analysis on resting-state epochs of routinely performed interictal EEG recordings. The diagnostic power was high: sensitivity and specificity of 0.96 and 0.95 respectively. The values obtained by the diagnostic model clearly exceed the sensitivity and specificity based on epileptiform EEG activity only, namely 0.77 and 0.91 respectively.

There is a rising interest in understanding brain functioning from a network perspective. However, only few studies have aimed to explore the clinical value of these functional networks in epilepsy. For example, two studies have used a functional network approach to predict post-surgical outcome in epilepsy surgery. Wilke and colleagues studied invasive corticography recordings during epilepsy surgery to correlate betweenness centrality, to the resected cortical regions. They found that the betweenness centrality was correlated with the resected areas of patients who became seizure-free after surgery [38]. Yet another study, found that functional connectivity in patients with seizure-recurrence after epilepsy surgery was less lateralized compared to those who were seizure free [39], [40]. Although these results are promising, the study domain has been limited to epilepsy surgery and data acquisition has been applied to specific patients only.

This study was undertaken to bridge the gap between the field of more fundamental computational neuroscience and daily clinical practice. The use of network characteristics in diagnosing epilepsy extends the method used in a recent study [13]. Douw and colleagues used EEG functional connectivity to diagnose epilepsy after a first suspected seizure and found a specificity of 0.76 and sensitivity of 0.62. The higher sensitivity and specificity values obtained in our study argue for a multivariable prediction design and machine learning models, such as the random forest classifier, although one may argue that the high sensitivity and specificity may be partly due to the homogeneity of our patients. Douw and colleagues found the theta frequency band to be most sensitive in terms of prediction. In our study we found excellent results using the broadband frequency, suggesting that the discriminatory power is not restricted to one specific frequency band.

A potential limitation of our study is that the inclusion was restricted to children diagnosed with partial epilepsy. Hence, this prediction model can be applied in children with partial epilepsy only. In addition, selecting resting-state EEG epochs in very young children can be challenging since EEG recordings may have motion-induced artifacts. Clear instructions and longer registrations could potentially overcome this limitation. Clearly, its clinical value will increase with extended inclusion of adults and other epilepsy syndromes.

Early accurate diagnosis is particularly valuable in young children to inform and guide parents, to prompt treatment decisions and to limit the period of uncertainty and unnecessary risks [1]. To explore the true diagnostic value of our proposed predictive model, larger studies are required, especially using external validation, although we corrected for too optimistic model results using stringent internal validation. The use of freely available software packages for model development validation should facilitate the process of external validation [29], [30], [31], [32], [37]. Our prediction model, based on routinely performed EEG, is appealing due to the immediate clinical availability. Further, standard EEG epoch selection is straightforward, network calculations are relatively fast and software is freely available. Although current selection of epochs was performed manually, software is available to reduce the time spent to select ‘resting state’ epochs semi-automatically [41].

Indications for future research are multiple. Use of other modalities such as resting-state functional magnetic resonance imaging, high density EEG and magnetoencephalogram may lead to development of diagnostic models with even higher accuracy due to their superior spatial resolution of functional networks.

In conclusion, although more and larger studies are needed, this study clearly shows that functional network characteristics are promising and clinically useful in the early diagnosis of partial epilepsy in children after their first seizure(s).

## Supporting Information

Table S1 **Clinical characteristics of 35 children with new onset partial epilepsy.**
(DOCX)

Table S2 **Clinical characteristics of 35 controls.**
(DOCX)
