# Supplementary material for: Improved Diagnosis in Children with Partial Epilepsy Using a Multivariable Prediction Model Based on EEG Network Characteristics
Source: PLoS One. 2013 Apr 2;8(4):e59764. doi: 10.1371/journal.pone.0059764 (PMC3614973; doi:10.1371/journal.pone.0059764)
Supplement: Table S1 — Clinical characteristics of 35 children with new onset partial epilepsy. (DOCX) [file pone.0059764.s001.docx]

|  | **Gender**  **(Female/Male)** | **Age (years)**  **(at presentation)** | **Number of seizures  (prior to referral)** | **diagnosis** | **EEG report** | **Neuroimaging report** |
| --- | --- | --- | --- | --- | --- | --- |
| 1 | Female | 4.6 | 2 | Idiopathic epilepsy | IEDs in left central region | No abnormalities |
| 2 | Male | 5.1 | 1 | Crypotogenic epilepsy | Bilateral IEDs in occipital region | No abnormalities |
| 3 | Female | 5.1 | 36 | Crypotogenic epilepsy | IEDs in right central region | No abnormalities |
| 4 | Male | 5.3 | 1 | Idiopathic epilepsy | Abnormal slowing in right central region | No abnormalities |
| 5 | Female | 6.2 | 1 | Idiopathic epilepsy | Spikes in left frontopolar region | No abnormalities |
| 6 | Female | 6.8 | unknown | Symptomatic epilepsy | IEDs and spikes in left occipital region | Small hyperintense lesions in occiptal region |
| 7 | Male | 7.0 | 3 | Idiopathic epilepsy | IEDs in left central region | No abnormalities |
| 8 | Female | 7.1 | 5 | Idiopathic epilepsy | IEDs and spikes in right centrotemporal region | No abnormalities |
| 9 | Male | 7.2 | 1 | Symptomatic epilepsy | No abnormalities | Several hyperintense lesions in basal ganglia |
| 10 | Male | 7.4 | 1 | Idiopathic epilepsy | IEDs in left occipital region | No abnormalities |
| 11 | Male | 7.9 | 1 | Idiopathic epilepsy | IEDs in left centrotemporal region | No abnormalities |
| 12 | Female | 8.4 | 1 | Idiopathic epilepsy | IEDs and spikes in right centrotemporal region | No abnormalities |
| 13 | Male | 9.0 | 4 | Idiopathic epilepsy | Spikes in left central region | No abnormalities |
| 14 | Male | 9.0 | 1 | Idiopathic epilepsy | IEDs in left parietal region | No abnormalities |
| 15 | Male | 9.2 | 1 | Crypotogenic epilepsy | IEDs in left parietal region | No abnormalities |
| 16 | Male | 9.2 | 5 | Crypotogenic epilepsy | No abnormalities | No abnormalities |
| 17 | Female | 9.4 | unknown | Crypotogenic epilepsy | Spikes in left temporal region | Small subcortical lesion in cerebellum |
| 18 | Male | 9.6 | 30 | Crypotogenic epilepsy | No abnormalities | Several perivascular abnormalities |
| 19 | Male | 9.6 | 1 | Idiopathic epilepsy | No abnormalities | No neuroimaging available |
| 20 | Male | 9.9 | 2 | Idiopathic epilepsy | Bilateral spikes in centroparietal region | No abnormalities |
| 21 | Male | 10.4 | 1 | Symptomatic epilepsy | Abnormal slowing and spikes in right centroparietal region | Several (sub)cortical lesions |
| 22 | Female | 10.8 | unknown | Crypotogenic epilepsy | No abnormalities | No abnormalities |
| 23 | Female | 12.1 | 3 | Symptomatic epilepsy | Abnormal slowing in left frontal region | Arachnoidal cyst in left temporal lobe |
| 24 | Male | 12.3 | 15 | Symptomatic epilepsy | No abnormalities | Gliosis and atrophy in left parietal lobe |
| 25 | Male | 12.5 | 2 | Crypotogenic epilepsy | No abnormalities | No abnormalities |
| 26 | Male | 12.9 | 7 | Idiopathic epilepsy | Bilateral IEDs in temporal regions | Small cortical dysplasia in left frontal/insular region |
| 27 | Male | 13.1 | 3 | Crypotogenic epilepsy | Abnormal slowing and IED in right frontopolar region | No abnormalities |
| 28 | Male | 13.3 | 1 | Idiopathic epilepsy | Spikes and IED in temporal region | No neuroimaging available |
| 29 | Male | 13.3 | unknown | Symptomatic epilepsy | IEDs in left temporal region | Left hippocampus atrophy |
| 30 | Female | 14.0 | 10 | Symptomatic epilepsy | IEDs in right temporal region | Right temporal arteriovenous malformation |
| 31 | Male | 14.2 | 2 | Crypotogenic epilepsy | IEDs in left frontopolar region | Several hyperintense lesions |
| 32 | Male | 15.3 | 2 | Crypotogenic epilepsy | Abnormal slowing and spikes in left temporal region | No abnormalities |
| 33 | Male | 15.3 | unknown | Crypotogenic epilepsy | Spikes in left frontotemporal region | No abnormalities |
| 34 | Female | 15.4 | 2 | Symptomatic epilepsy | Spikes in midtemporal region | No neuroimaging available |
| 35 | Male | 16.8 | 2 | Crypotogenic epilepsy | No abnormalities | No abnormalities |

**Improved diagnosis in children with partial epilepsy using a multivariable prediction model based on EEG network characteristics**

**Table S1:** Clinical characteristics of 35 children with new onset partial epilepsy. IED = Interepileptic discharges
