# Supplementary material for: Improved Diagnosis in Children with Partial Epilepsy Using a Multivariable Prediction Model Based on EEG Network Characteristics
Source: PLoS One. 2013 Apr 2;8(4):e59764. doi: 10.1371/journal.pone.0059764 (PMC3614973; doi:10.1371/journal.pone.0059764)
Supplement: Table S2 — Clinical characteristics of 35 controls. (DOCX) [file pone.0059764.s002.docx]

**Improved diagnosis in children with partial epilepsy using a multivariable prediction model based on EEG network characteristics**

**Table S2:** Clinical characteristics of 35 controls

|  | **Gender**  **(Female/Male)** | **Age (years)**  **(at presentation)** | **diagnosis** | **EEG report** |
| --- | --- | --- | --- | --- |
| 1 | Female | 5.5 | Syncope | No abnormalities |
| 2 | Male | 5.4 | Staring/non-attendance | No abnormalities |
| 3 | Female | 5.5 | Breath holding spells | No abnormalities |
| 4 | Male | 6.1 | Behavioural/psychological | No abnormalities |
| 5 | Female | 6.1 | Behavioural/psychological | No abnormalities |
| 6 | Female | 6.1 | Night terrors | No abnormalities |
| 7 | Male | 6.7 | Staring/non-attendance | Abnormal slowing in temporal regions |
| 8 | Female | 7.3 | Night terrors | No abnormalities |
| 9 | Male | 7.1 | Behavioural/psychological | No abnormalities |
| 10 | Male | 7.3 | Behavioural/psychological | No abnormalities |
| 11 | Male | 8.2 | Behavioural/psychological | No abnormalities |
| 12 | Female | 8.5 | Night terrors | No abnormalities |
| 13 | Male | 8.3 | Syncope | No abnormalities |
| 14 | Male | 8.6 | Behavioural/psychological | No abnormalities |
| 15 | Male | 8.8 | Behavioural/psychological | No abnormalities |
| 16 | Male | 9.2 | Syncope | No abnormalities |
| 17 | Female | 8.9 | Behavioural/psychological | No abnormalities |
| 18 | Male | 9.4 | Arrhythmia | No abnormalities |
| 19 | Male | 9.7 | Staring/non-attendance | No abnormalities |
| 20 | Male | 9.7 | Behavioural/psychological | No abnormalities |
| 21 | Male | 10.1 | Syncope | No abnormalities |
| 22 | Female | 11.5 | Behavioural/psychological | No abnormalities |
| 23 | Female | 11.8 | Staring/non-attendance | Abnormal slowing and aspecific spikes in central regions |
| 24 | Male | 10.2 | Staring/non-attendance | No abnormalities |
| 25 | Male | 11.5 | Staring/non-attendance | No abnormalities |
| 26 | Male | 11.6 | Syncope | No abnormalities |
| 27 | Male | 11.9 | Syncope | No abnormalities |
| 28 | Male | 12.2 | Staring/non-attendance | No abnormalities |
| 29 | Male | 12.6 | Syncope | Aspecific spikes in frontal regions |
| 30 | Female | 14.5 | Syncope | No abnormalities |
| 31 | Male | 12.9 | Behavioural/psychological | No abnormalities |
| 32 | Male | 14.6 | Syncope | No abnormalities |
| 33 | Male | 15.6 | Syncope | No abnormalities |
| 34 | Female | 15.5 | Segmental myclonus | No abnormalities |
| 35 | Male | 15.8 | Syncope | No abnormalities |
